# Comparison of Treatment Regimens in Management of Severe Hypercalcemia Due to Vitamin D Intoxication in Children

**DOI:** 10.4274/jcrpe.galenos.2018.2018.0131

**Published:** 2019-05-28

**Authors:** Korcan Demir, Hakan Döneray, Cengiz Kara, Zeynep Atay, Semra Çetinkaya, Atilla Çayır, Ahmet Anık, Erdal Eren, Ahmet Uçaktürk, Gülay Can Yılmaz, Ayça Törel Ergür, Mustafa Kendirci, Zehra Aycan, Abdullah Bereket, Murat Aydın, Zerrin Orbak, Behzat Özkan

**Affiliations:** 1 Dokuz Eylül University Faculty of Medicine, Department of Pediatric Endocrinology, İzmir, Turkey; 2 Atatürk University Faculty of Medicine, Department of Children’s Health and Disease, Erzurum, Turkey; 3 Ondokuz Mayıs University Faculty of Medicine, Department of Children’s Health and Disease, Samsun, Turkey; 4 Marmara University Faculty of Medicine, Department of Pediatric Endocrinology, İstanbul, Turkey; 5 University of Health Sciences, Dr. Sami Ulus Obstetrics and Gynecology, Children’s Health and Disease, Health Implementation and Research Center, Ankara, Turkey; 6 Erzurum State Training and Research Hospital, Clinic of Pediatric Endocrinology, Erzurum, Turkey; 7 Adnan Menderes University Faculty of Medicine, Department of Children’s Health and Disease, Aydın, Turkey; 8 Uludağ University Faculty of Medicine, Department of Pediatric Endocrinology, Bursa, Turkey; 9 Ankara Children’s Hematology and Oncology Training Hospital, Clinic of Pediatric Endocrinology, Ankara, Turkey; 10 Kırıkkale University Faculty of Medicine, Department of Children’s Health and Disease, Kırıkkale, Turkey; 11 Erciyes University Faculty of Medicine, Department of Pediatric Endocrinology, Kayseri, Turkey; 12 Yıldırım Beyazıt University Faculty of Medicine, Department of Pediatric Endocrinology, Ankara, Turkey; 13 University of Health Sciences Dr. Behçet Uz Children’s Training and Research Hospital, Clinic of Pediatric Endocrinology, İzmir, Turkey

**Keywords:** Nutrition, rickets, stoss therapy, steroid, over-the-counter drugs

## Abstract

**Objective:**

No large study has been conducted to date to compare the effectiveness of prednisolone, alendronate and pamidronate as first-line treatment in children with hypercalcemia due to vitamin D intoxication. The aim was to perform a multicenter, retrospective study assessing clinical characteristics and treatment results.

**Methods:**

A standard questionnaire was uploaded to an online national database system to collect data on children with hypercalcemia (serum calcium level >10.5 mg/dL) due to vitamin D intoxication [serum 25-hydroxyvitamin D (25(OH)D) level >150 ng/mL] who were treated in pediatric endocrinology clinics.

**Results:**

Seventy-four children [median (range) age 1.06 (0.65-1.60) years, 45 males (61%) from 11 centers] were included. High-dose vitamin D intake was evident in 77% of the cases. At diagnosis, serum calcium, phosphorus, alkaline phosphatase, 25(OH)D and parathyroid hormone concentrations were 15±3.2 mg/dL, 5.2±1.2 mg/dL, 268±132 IU/L, 322 (236-454) ng/mL, and 5.5 (3-10.5) pg/mL, respectively. Calcium levels showed moderate correlation with 25(OH)D levels (r_s_=0.402, p<0.001). Patients were designated into five groups according to the initial specific treatment regimens (hydration-only, prednisolone, alendronate, pamidronate, and combination). Need for another type of specific drug treatment was higher in children who initially received prednisolone (p<0.001). Recurrence rate of hypercalcemia was significantly lower in children who were treated with pamidronate (p=0.02).

**Conclusion:**

Prednisolone is less effective in the treatment of children with severe hypercalcaemia secondary to vitamin D intoxication and timely implementation of other treatment regimens should be considered.

**What is already known on this topic?**

There are various treatment options for hypercalcemia. Pamidronate treatment efficiently lowers serum calcium levels in children with hypercalcemia due to vitamin D intoxication.

**What this study adds?**

To our knowledge, this study is the first to compare first-line treatment options for hypercalcemia due to vitamin D intoxication. Children receiving prednisolone for severe hypercalcemia often require another type of drug treatment. Pamidronate treatment prevents recurrence of hypercalcemia.

## Introduction

Vitamin D exerts significant effects on intestinal absorption of calcium and phosphorus, renal reabsorption of calcium and mineralization of bone. The primary source of vitamin D in humans is its synthesis in the skin, which requires adequate sunlight exposure, since vitamin D content of most foods is low. Clinical problems associated with vitamin D metabolism are mostly due to its deficiency and, accordingly, several guidelines exist for evaluation and management of vitamin D deficiency (1,2,3). However, pediatricians are also encountering children with mild-to-severe consequences of vitamin D intoxication, often associated with hypercalcemia. Vitamin D intoxication is generally defined as serum levels of 25-hydroxyvitamin D [25(OH)D] above 100-150 ng/mL (250-375 nmol/L) (1,3,4,5). Possible causes include treatment of vitamin D-deficient rickets with single or daily high doses of vitamin D (6,7), manufacturing errors of over-the-counter drugs (8,9), parental dosing errors (10), over-fortification of milk (11) and prescription of vitamin D without prior measurement of its serum level or without a definite diagnosis of rickets (12,13,14,15).

Treatment options for vitamin D intoxication in children currently include discontinuation of vitamin D intake, intravenous hydration (IH) with normal saline, administration of F, glucocorticoids, calcitonin, alendronate, pamidronate and hemodialysis. These practises are mostly based on case reports and small studies (4,5,7,8,9,10,12,13,15,16,17,18,19,20,21).

We aimed to assess the clinical characteristics of children with vitamin D intoxication in a multicenter, retrospective study. A further aim was to compare the results of different first-line treatment schedules in a large sample.

## Methods

A standard questionnaire was established in an online national database system (formerly www.favorsci.org, and currently http://cedd.saglik-network.org/) to collect clinical and laboratory data on children with hypercalcemia (serum calcium level >10.5 mg/dL) due to vitamin D intoxication [concurrent serum 25(OH)D level >150 ng/mL] who were treated in pediatric endocrinology clinics. The data were collected by a single nominated pediatric endocrinologist per center, who was responsible for registering patients onto the online database. The study protocol was approved by the Institutional Ethical Review Board University of Health Sciences Dr. Behçet Uz Children’s Hospital, 2014-01). Informed consent was not taken from the parents of the patients, given the retrospective design of the study, for which the data were simply extracted from patient files.

Seventy-four patients from 11 tertiary referral centers were enrolled. Participating centers were located in five of the seven geographical regions of Turkey. Forty of the cases had been previously reported elsewhere (8,12,14,19,20). All biochemical evaluations were performed in a standard laboratory setting. Hypercalcemia was classified according to the following serum calcium levels as: mild (10.5-11.9 mg/dL); moderate (12-14 mg/dL); and (severe >14 mg/dL) (22). Hypercalciuria was defined when spot urine calcium/creatinine ratio exceeded the upper limits of normal calcium excretion for different age groups: ≤6 months of age, >0.8; 7-12 months of age, >0.6; 1-3 years of age, >0.53; 3-5 years of age, >0.39; 5-7 years of age, >0.28; >7 years of age, >0.21 (23).

Firstly, the patients were assessed by their clinical characteristics of vitamin D intoxication. Secondly, patients were designated into groups according to the specific treatment type they had received in the first 48 hours, as shown below:

Group 1 (n=25): Oral hydration (OH) or IH ± furosemide (F)

Group 2 (n=9): IH ± F + prednisolone

Group 3 (n=11): IH + F + alendronate

Group 4 (n=21): IH + F + pamidronate

Group 5 (n=8): IH + F + prednisolone + pamidronate ± alendronate

Primary outcome measures related to treatment efficacy included: a) need for another specific drug treatment; b) elapsed time to achieve normocalcemia (8.5-10.5 mg/dL); and c) recurrence of hypercalcemia (elevation of calcium levels above >10.5 mg/dL after achievement of normocalcemia).

Secondary outcome measures were clinical features of, and factors associated with, hypercalcemia in children with vitamin D intoxication.

### Statistical Analysis

The data were statistically analyzed using Statistical Package for the Social Sciences Software, version 15.0 (IBM Inc., Chicago, Illinois, USA). Descriptive analyses were performed for all data sets. Depending on the distribution type of the variables, Pearson or Spearman correlation analysis was performed to detect the factors associated with serum calcium levels at the time of admission. Subsequently, variables associated with serum calcium levels at the time of admission were entered into a multiple linear regression analysis. The least explanatory covariates were consecutively removed from the model in a backward stepwise elimination method. Two separate Kruskal-Wallis tests were performed for comparison of non-parametric numerical data between groups 1-5 and 2-4. Chi-square or Fisher’s exact test (if expected count was below 5 in any of the cells) was used to compare categorical variables. All data were presented as n (%), mean ± standard deviation or median and interquartile range (IQR) minimum-maximum (range), where appropriate. Figures were prepared using GraphPad Prism version 6.01 for Windows (GraphPad Software, La Jolla, California, USA, www.graphpad.com).

## Results

The study group included 74 children who were treated for vitamin D intoxication between the years 2002 and 2014. The median age of the subjects was 1.06 (IQR 0.65-1.60; range 0.04-7.38) years and 45 were male (60.8%) (Table 1). The median number of patients enrolled per center was 4 (IQR 2-7; range 1-27). Nearly half of the patients were younger than one year of age (n=33, 44.6%). Twenty-one cases were between 1-2 years of age (39.2%) and 12 (16.2%) subjects were older than two years of age. Only seven (9.5%) of the cases had chronic illnesses (hypotonic infant, n=2; developmental dysplasia of the hip, n=2; meningomyelocele, n=1; wheezy infant, n=1; cerebral palsy and epilepsy, n=1). The most common presenting symptoms were vomiting (n=47, 63.5%), loss of appetite (n=35, 47.3%), and constipation (n=27, 36.5%). Five of the patients were asymptomatic and were incidentally found to have mild-to-moderate hypercalcemia (serum calcium levels, 10.8-13 mg/dL).

Approximately three-quarters of the patients (n=57, 77%) had a clear history of high-dose vitamin D intake [median dose, 600,000 (IQR 600,000-900,000; range 300,000-5,400,000) units. The majority of the patients (n=40, 70.2%) had received multiple doses of vitamin D on separate days due to accidental overdose by parents or overdose secondary to faulty dose. The median time from first dose of vitamin D to admission was 6.2 (IQR 3.6-9.4; range, 1.5-67.1) weeks (Table 1). The most common reason for vitamin D use was presumptive diagnosis of vitamin D deficiency, based on non-specific complaints including delay in walking or eruption of teeth without proper evaluation (n=41, 71.9%). Active rickets was the reason for vitamin D treatment in only three cases (5.3%, serum calcium levels, 10.8, 10.8, and 15 mg/dL, with the latter being due to parental dosing error).

Serum calcium, phosphorus, alkaline phosphatase (ALP), 25(OH)D and parathyroid hormone (PTH) concentrations of the study group were as follows: 15±3.2 mg/dL (range, 10.8-23.5), 5.2±1.2 mg/dL (range, 2.48-7.7), 268±132 IU/L (range, 89-652), 322 (IQR 236-454, range 150-1978) ng/mL, 5.5 (IQR 3.0-10.5, range 0.5-38.0) pg/mL, respectively (see Table 1). The majority of the patients (n=43, 58.1%) had severe hypercalcemia (>14 mg/dL), normal phosphate values in 55/69 (79.7%) cases with available data and suppressed PTH in 51/65 (78.4%) cases with available data. At the time of admission, hypercalciuria and nephrocalcinosis and/or nephrolithiasis were found in 46/57 (81%) of cases with available data and 33/68 patients (48.5%) of cases with available data, respectively.

**Figure 1:**
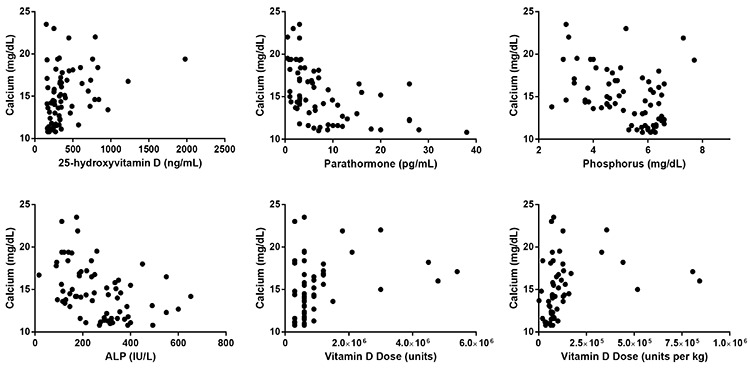
Correlation analyses of various variables with calcium and 25-hydroxyvitamin D levels at the time of admission ALP: alkaline phosphatase

Serum calcium concentrations at onset showed a moderate negative correlation with serum PTH (n=65, r_s_=-0.588, p<0.001) and weak or moderate correlations with serum concentrations of 25(OH)D (n=74, r_s_=0.402, p<0.001), phosphorus (n=69, r=-0.379, p=0.001), ALP (n=66, r=-0.416, p=0.001), vitamin D dose (n=57, r_s_=0.383, p=0.004), and vitamin D dose per kilogram of body weight (n=57, r_s_=0.483, p<0.001) (Figure 1). However, spot urine calcium/creatinine ratio (n=57, r=-0.095, p=0.484) and time to admission from first dose of vitamin D (n=57, r=-0.169, p=0.235) showed no correlation with serum calcium levels. In the multiple linear regression analysis including age, vitamin D dose, vitamin D dose per kilogram of body weight, time to admission from first dose of vitamin D and serum levels of 25(OH)D, the final model contained two baseline variables which were independently associated with serum calcium levels. These were serum levels of 25(OH)D [B=0.005 (95% CI 0.02, 0.008), p=0.001] and vitamin D dose (per 100,000 IU) [B=0.089 (95% CI 0.023, 0.155), p=0.009]. These two variables together explained 22.6% of the variance of serum calcium levels [R2=0.226, F(4)=9.327, p<0.001].

**Figure 2:**
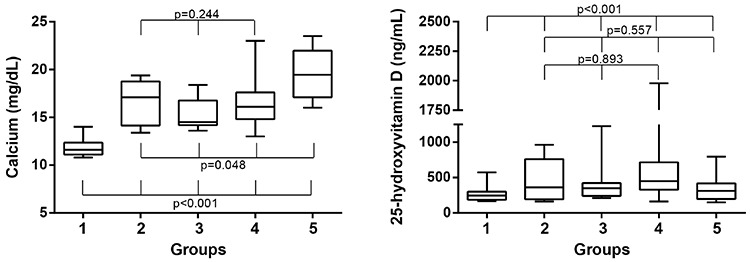
Box-whisker graphs of serum calcium and 25-hydroxyvitamin D levels among the groups (the horizontal lines within the boxes indicate the median, boundaries of the boxes indicate the 25th and 75th percentiles, and the whiskers indicate the highest and lowest values of the results)

Patients were designated into five groups according to their specific treatment regimens in the first 48 hours (Table 2). None of the patients had renal failure or required hemodialysis. Vitamin D intake and serum levels of calcium, phosphorus, ALP, 25(OH)D and PTH were significantly different among groups 1-5 (Table 2). We hypothesized that calcium and 25(OH)D levels at the time of admission should be similar among groups to make a reliable comparison regarding treatment efficiency. Figure 2 shows that only groups 2, 3, and 4 met this criterion.

The data regarding treatments and outcomes are shown in Table 3. Type and volume of hydration fluid, dose and duration of F treatment were similar among groups 2, 3, and 4. Six subjects (66.7%) in group 2 required another specific drug treatment after the first 48 hours of admission (one patient, pamidronate and calcitonin on day 10; two patients, pamidronate on days 3 and 4; three patients, calcitonin) while this was the case for one patient (4.8%) in group 4 (prednisolone, starting from day 6 of treatment) and none in group 3 (p<0.001). The time to achieve normocalcemia was comparable (p=0.099) among groups 2, 3, and 4. However, recurrence rate of hypercalcemia was significantly lower in group 4 compared to groups 2 and 3 [0 (0%), 2 (25%), and 3 (30%), respectively, p=0.02]. Sixty-four of 68 subjects with initial renal sonograms were reassessed during follow-up and the ratio of nephrocalcinosis and/or nephrolithiasis was found to have decreased to 28.1% (n=18) after a follow-up duration of 1±0.9 years. The distribution was not significantly different among groups 2, 3, and 4 (p=0.268).

## Discussion

The majority of the children in our study group were younger than two years of age and did not have a pre-existing chronic health condition. Their pretreatment serum 25(OH)D levels were unknown. The upper limit of daily oral intake of vitamin D for healthy children aged <1 and 1-3 years are reported as 1000-1500 IU and 2000-2500 IU, respectively (1). In the present study, minimum and mean doses of vitamin D intake that led to hypercalcemia were 300,000 IU and 1,020,000 IU, respectively. In one study, treatment with 300,000 IU of vitamin D in 3 to 36-month-old subjects with nutritional vitamin D deficiency rickets (n=20) was reported to cause hypercalcemia in two patients (10%) (24). On the other hand, it was reported that calcium levels did not exceed the upper limit after treatment with the same vitamin D dose in 32 children aged between 3-17 years with vitamin D deficiency/insufficiency (25). In addition, vitamin D dose (both total and per kg of body weight) and serum 25(OH)D levels in our study were only moderately correlated with the degree of hypercalcemia. Dietary calcium intake and existence of conditions leading to vitamin D hypersensitivity might contribute to development and severity of hypercalcemia associated with vitamin D intoxication (1,3,26).

Treatment is warranted in vitamin D intoxication, as resulting hypercalcemia is associated with mild-to-severe gastrointestinal, renal, central nervous system, cardiovascular, musculoskeletal, ophthalmological, and skin complications (4). The most common symptoms in our series were related with the gastrointestinal system including vomiting (63.5%), loss of appetite (47.3%) and constipation (36.5%). The most common clinical finding was nephrocalcinosis and/or nephrolithiasis (48.5%). Various studies have demonstrated that the majority of symptoms associated with vitamin D-induced nephrocalcinosis persist for years (27,28). In the present study, nephrocalcinosis and/or nephrolithiasis disappeared in nearly half of the affected cases.

Currently, there are various treatment regimens for vitamin D intoxication. A report including 11 adults from 1948 indicates that the only available methods at that time were elimination of vitamin D, low calcium diet and OH. Howard and Meyer (29) reported that the shortest time to achieve normocalcemia was 3-12 weeks in four subjects (36.3%, calcium levels 12.4-14.9 mg/dL) while it took over a year in three cases (27.2%, calcium levels 13.7-14.9 mg/dL). In the present study, a similar treatment was applied in group 1 [median (range) calcium level 11.6 mg/dL (11.1-12.4)]. In addition, intravenous fluids and F were also used. Both additional therapies as well as milder degree of hypercalcemia at presentation resulted in a much shorter time to reach normocalcemia.

Other treatment regimens for vitamin D intoxication include calcitonin, prednisolone, alendronate, pamidronate and hemodialysis (3,4). Glucocorticoids decrease both renal reabsorption and intestinal absorption of calcium. However, their onset of action may take up to three days (3). Hatun and Cizmecioğlu (16) noted that normocalcemia could not be achieved and bisphosphonates were needed after over one month of glucocorticoid treatment in two infants with vitamin D intoxication (calcium levels at the time of admission, 14.9 and 18 mg/dL). Sezer et al (13) reported that four infants with vitamin D intoxication were given prednisolone (2 mg/kg/d) initially and two of them (calcium levels at the time of admission 16.5 and 19.1 mg/dL) required further alendronate treatment due to persistence of hypercalcemia after 15 and 23 days. Kara et al (15) reported that three children who were given prednisolone (1 mg/kg/d) for vitamin D intoxication (calcium levels at the time of admission: 16.0, 16.7, and, 19.7 mg/dL) reached normocalcemia after 12-26 days but that the hypercalcemia recurred in all of them after discontinuation of treatment. In the present study, nine patients with median calcium and 25(OH)D levels of 17.1 mg/dL and 361 ng/mL, respectively, were started on prednisolone [group 2, median (range) dose of 1 (1-2) mg/kg/day for 5 (3-10) days] as first-line treatment. However, two-thirds of these patients required another specific drug treatment due to persistence of the hypercalcemia and recurrence rate in this group was 25%. These data, together with the published evidence, indicate that prednisolone treatment has a low efficiency in “severe” hypercalcemia.

Bisphosphonates can lower calcium levels in subjects with vitamin D intoxication via their antiresorptive effect on bones (3). Alendronate as a first-choice treatment for vitamin D intoxication was first reported by Bereket and Erdogan (17) in 2003 in a 3-month-old infant with a serum calcium level of 18.5 mg/dL. A total of 30 mg of alendronate was given between the second and sixth days of treatment, resulting in normocalcemia. Orbak et al (21) reported a 7-year-old male child who was given 4,500,000 units of vitamin D for suspected vitamin D deficiency. Alendronate treatment was started at a serum calcium level of 14.8 mg/dL and normocalcemia was achieved by the 15th day after a cumulative dose of 45 mg that was given in five doses, 2-7 days apart. Sezer et al (13) described two subjects (serum calcium levels at the time of admission, 15.2 and 17 mg/dL) who were given a single dose of 10 mg of aledronate (13). Calcium levels returned to normal after five days and did not increase afterwards. Kara et al (15) reported two cases (serum calcium levels at the time of admission, 13.7 and 16.9 mg/dL) for whom alendronate (10 mg/d for seven consecutive days) was used directly. Normocalcemia was achieved after 3 and 4 days and no recurrence was reported. In the present study eleven patients in group 3, with a median (range) calcium concentration of 14.5 (14.2-16.8) mg/dL, received alendronate at a median dose of 6.7 mg [median (range) number of administrations, 3 (1-10)]. Although none of the cases required another specific drug treatment, hypercalcemia recurred in three patients.

The first experience with pamidronate, an intravenously given bisphosphonate, for vitamin D intoxication in children was reported by Ezgu et al (18) in 2004. The patient was a 3-month-old infant and was first treated with prednisolone. Four doses of pamidronate (0.2 mg/dose) were needed to achieve normocalcemia. Kara et al (15) reported pamidronate use as the first-line treatment in 13 children with vitamin D intoxication in whom median (range) calcium level at the time of admission was 16.5 (13.6-18.8) mg/dL. The first dose of pamidronate was 1 mg/kg when serum calcium levels were between 12-15 mg/dL and 2 mg/kg for levels above 15 mg/dL. Only two cases required a second pamidronate dose and none of them required prednisolone or alendronate with no recurrence being observed. In the present study, 21 children in group 4 with a median (range) calcium level 16.1 (14.8-17.6) mg/dL received pamidronate (median dose 1 mg/kg) as first-choice treatment. Similarly, none of the subjects required an alternative drug treatment or experienced recurrent hypercalcemia.

There exist two studies comparing the consequences of different treatments. Sezer et al (13) noted the superiority of alendronate (n=4) compared to prednisolone (n=4) and Kara et al (15) reported superiority of pamidronate (n=18) to prednisolone (n=6) and alendronate (n=3). However, in both studies, some of the children had received other treatment regimens previously. In the present study which comprised a larger cohort, we were able to group the subjects according to the first-line treatments only. In mild hypercalcemia (group 1), oral or IH and F were sufficient to achieve normocalcemia. For very severe hypercalcemia, physicians tended to use combination therapies as first-line treatment (group 5). Groups 2, 3, and 4 had similar patient characteristics and serum calcium and 25(OH)D levels enabling us to compare the consequences of prednisolone (group 2), alendronate (group 3) and pamidronate (group 4) treatments. Pamidronate as a first-line treatment resulted in shorter duration of IH and no recurrence of hypercalcemia. Prednisolone treatment was not as effective as other regimens in lowering serum calcium levels and the majority of children who were given prednisolone subsequently required another specific drug treatment in order to achieve normocalcemia.

### Study Limitations

There were some limitations associated with our study. Numbers of reported patients were not similar in the different centers which contributed to the study. Over representation of one center in a particular treatment group might have influenced other unmeasured factors including variability in laboratory measurements that could affect outcomes. In addition, lower number of cases in group 2 (n=9) compared to group 4 (n=21) might have affected our judgements regarding the efficiency of prednisolone. However, as discussed above, there are many case reports in the medical literature supporting our findings.

## Conclusion

In conclusion, evaluation of this largest cohort of pediatric vitamin D intoxication resulting in hypercalcemia suggests that cases with serum calcium levels below 12 mg/dL can be treated without prednisolone and bisphosphonates. Prednisolone treatment is less effective in the treatment of children with “severe” hypercalcemia (serum calcium levels above 14 mg/dL) and prompt implementation of pamidronate should be considered.

## Figures and Tables

**Table 1:**
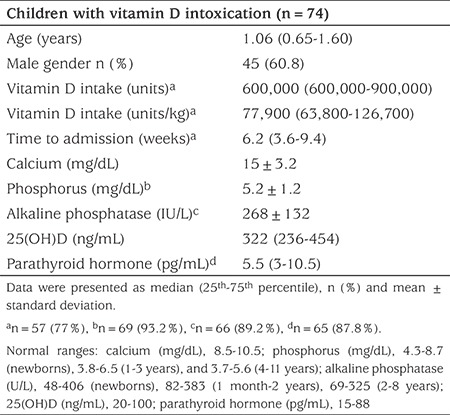
Characteristics of the total group of subjects at admission

**Table 2:**
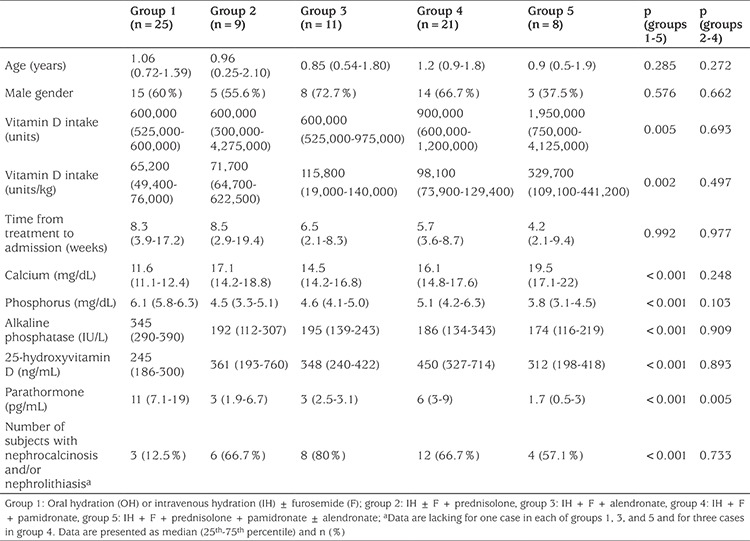
Characteristics of the patients among the groups at admission

**Table 3:**
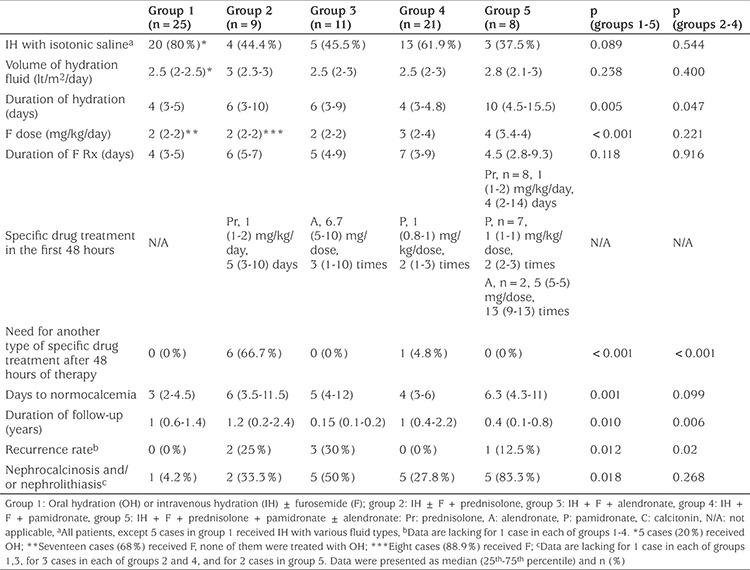
Treatment characteristics of the patients among the groups
